# Supplementary material for: Preclinical development of kinetin as a safe error-prone SARS-CoV-2 antiviral able to attenuate virus-induced inflammation
Source: Nat Commun. 2023 Jan 13;14:199. doi: 10.1038/s41467-023-35928-z (PMC9837764; doi:10.1038/s41467-023-35928-z)
Supplement: Supplementary file 1 — Supplementary Information [file 41467_2023_35928_MOESM1_ESM.pdf]

Supplementary Figures and Tables

Preclinical development of kinetin as a safe error-prone SARS-CoV-2 antiviral able to attenuate virus-induced inflammation

Thiago M. L. Souza[1,2,*,#], Vagner D. Pinho[3], Cristina F. Setim[4]; Carolina Q. Sacramento[1,2], Rodrigo Marcon[4], Natalia Fintelman-Rodrigues[1,2], Otavio A. Chaves[1,2], Melina Heller[4], Jairo R. Temerozo[1,5,6], André C. Ferreira[1,2,7], Mayara Mattos[1,2], Patrícia B. Momo[3], Suelen S. G. Dias[1], João Gesto[1,2], Filipe Pereira-Dutra[1], João P. B. Viola[8], Celso Martins Queiroz-Junior[9], Lays Cordeiro Guimarães[10], Ian Meira Chaves[9], Pedro Pires Goulart Guimarães[10], Vivian Vasconcelos Costa[9], Mauro Martins Teixeira[9], Dumith Chequer Bou-Habib[5,6], Patrícia T. Bozza[1], Anderson R. Aguillón[3], Jarbas Siqueira-Junior[4], Sergio Macedo-Junior[4], Edineia L. Andrade[4]; Guilherme P. Fadanni[4]; Sara E. L. Tolouei[4]; Francine B. Potrich[4]; Adara A. Santos[4], Naiani F. Marques[4]; João B. Calixto[4,*,#], Jaime A. Rabi[3,*,#]

[#]These authors contributed equally to this work

[1]Laboratório de Imunofarmacologia, Oswaldo Cruz Institute, Fundação Oswaldo Cruz (Fiocruz), Rio de Janeiro, RJ, Brazil

[2]National Institute for Science and Technology on Innovation in Diseases of Neglected Populations (INCT/IDPN), Center for Technological Development in Health (CDTS), Fiocruz, Rio de Janeiro, RJ, Brazil

[3]Microbiológica Química e Farmacêutuca, Doutor Nicanor, 238 Inhaúma, Rio de Janeiro, RJ, Brazil

[4]Centro de Inovação e Ensaios Pré-clínicos and National Institute for Science and Technology on Innovation in Medicines and Identification of New Therapeutics Targets (INCT-INOVAMED). Avenida Luiz Boiteux Piazza, 1302 Cachoeira do Bom Jesus, 88056-000 Florianópolis, SC Brazil

[5]National Institute for Science and Technology on Neuroimmunomodulation (INCT/NIM), Oswaldo Cruz Institut, Fiocruz, Rio de Janeiro, RJ, Brazil;

[6]Laboratório de Pesquisa sobre o Timo, Oswaldo Cruz Institute, Fiocruz, Rio de Janeiro, RJ, Brazil

[7]Universidade Iguaçu, Nova Iguaçu, RJ, Brazil

[8]Program of Immunology and Tumor Biology, Brazilian National Cancer Institute (INCA), Rua André Cavalcanti 37, 5th floor, Centro, Rio de Janeiro, Brazil

[9]Centro de Pesquisa e Desenvolvimento de Fármacos, Instituto de Ciências Biológicas, (ICB), Universidade Federal de Minas Gerais (UFMG), Minas Gerais, Brazil

[10]Department of Physiology and Biophysics, Institute of Biological Sciences, Universidade Federal de Minas Gerais, Belo Horizonte, MG, Brazil

***Corresponding authors**

Thiago Moreno L. Souza (thiago.moreno@fiocruz.br), João B. Calixto (joao.calixto@cienp.org.br), Jaime A. Rabi (jrabi@microbiologica.ind.br)**.**

A **MB-905**

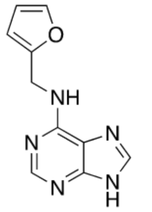

Chemical Formula: $C_{10}H_9N_5O$
Molecular Weight: 215.2160

B **MB-906**

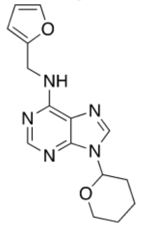

Chemical Formula: $C_{15}H_{17}N_5O_2$
Molecular Weight: 299.3340

C **MB-711**

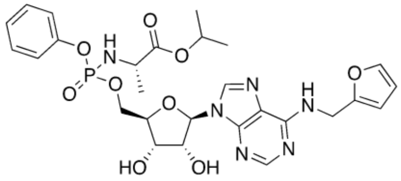

Chemical Formula: $C_{27}H_{33}N_6O_9P$
Molecular Weight: 616.5678

D **MB-801**

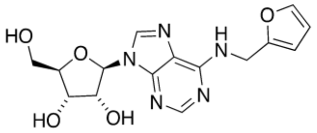

Chemical Formula: $C_{15}H_{17}N_5O_5$
Molecular Weight: 347.3310

Supplementary Fig. 1. Chemical structures of the original compounds used in this investigation.

A

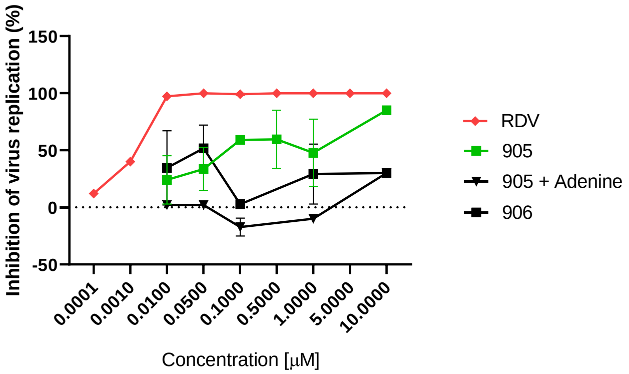

B

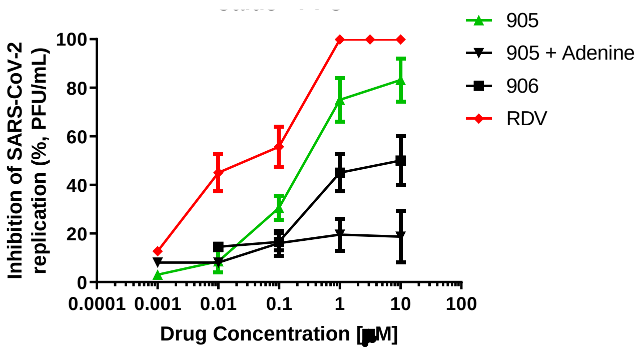

Supplementary Fig. 2. The anti-coronavirus activity of compound MB-905 requires the engagement of the enzyme adenine phosphoribosyl transferase (APRT). (**a**) HuH-7 cells, at a density of 5 x 10^4 cells/well in 96-well plates, were infected with SARS-CoV-2 at an MOI of 0.1 for 1 h at 37 °C and treated with the indicated concentrations of MB-905 in the presence or absence of 10 µM adenine or with its 9-tetrahydopyranyl derivative (MB-906). After 72 h, the cell monolayers were lysed, total RNA was extracted, and viral RNA synthesis was quantified by the detection of subgenomic RNA at the N region of the gene by real-time RT–PCR (n=3). (**b**)

Calu-3 cells (human type II pneumocytes), at a density of 5 x 10^4 cells/well in 96-well plates, were infected with SARS-CoV-2 at an MOI of 0.5 for 1 h at 37 °C and treated with the indicated concentrations of MB-905 in the presence or absence of 10 µM adenine or with MB-906 (n=3). After 48 h, cell supernatants were harvested, and infectious viral titers in the culture supernatant were measured by PFU/mL in Vero cells (**b**). The data represent the means ± SEMs of at least three independent experiments performed with three technical replicates per experiment.

Supplementary Table 1. In vitro pharmacological parameters of MB 905 and related compounds in inhibiting SARS-CoV-2 replication in Calu-3 cells.

	One treatment				Two treatments			
**Compounds**	EC_{50} [μM]	EC_{90} [μM]	CC_{50} [μM]	SI	EC_{50} [μM]	EC_{90} [μM]	CC_{50} [μM]	SI
**Remdesivir**	0.15 ± 0.03	0.4 ± 0.1	350 ± 50	2.300	0.01 ± 0.003	0.2 ± 0.01	330 ± 40	3,3000
**MK-4482**	0.72 ± 0.12	3.4 ± 0.5	58 ± 18	80	0.02 ± 0.008	8.6 ± 0.2	53 ± 12	2,900
**MB-905**	0.31 ± 0.05	2.8 ± 0.3	620 ± 80	2.000	0.03 ± 0.004	2.1 ± 0.3	538 ± 64	17,933
**MB-711**	1.1 ± 0.03	9.2 ± 0.1	562 ± 46	510	0.01 ± 0.008	3.8 ± 0.3	580 ± 72	58,000
**MB-801**	0.18 ± 0.02	7.2 ± 0.5	550 ±32	3.055	0.02 ± 0.003	3.7 ± 0.2	530 ± 65	26,500

SI – Selectivity index = CC_{50}/EC_{50}

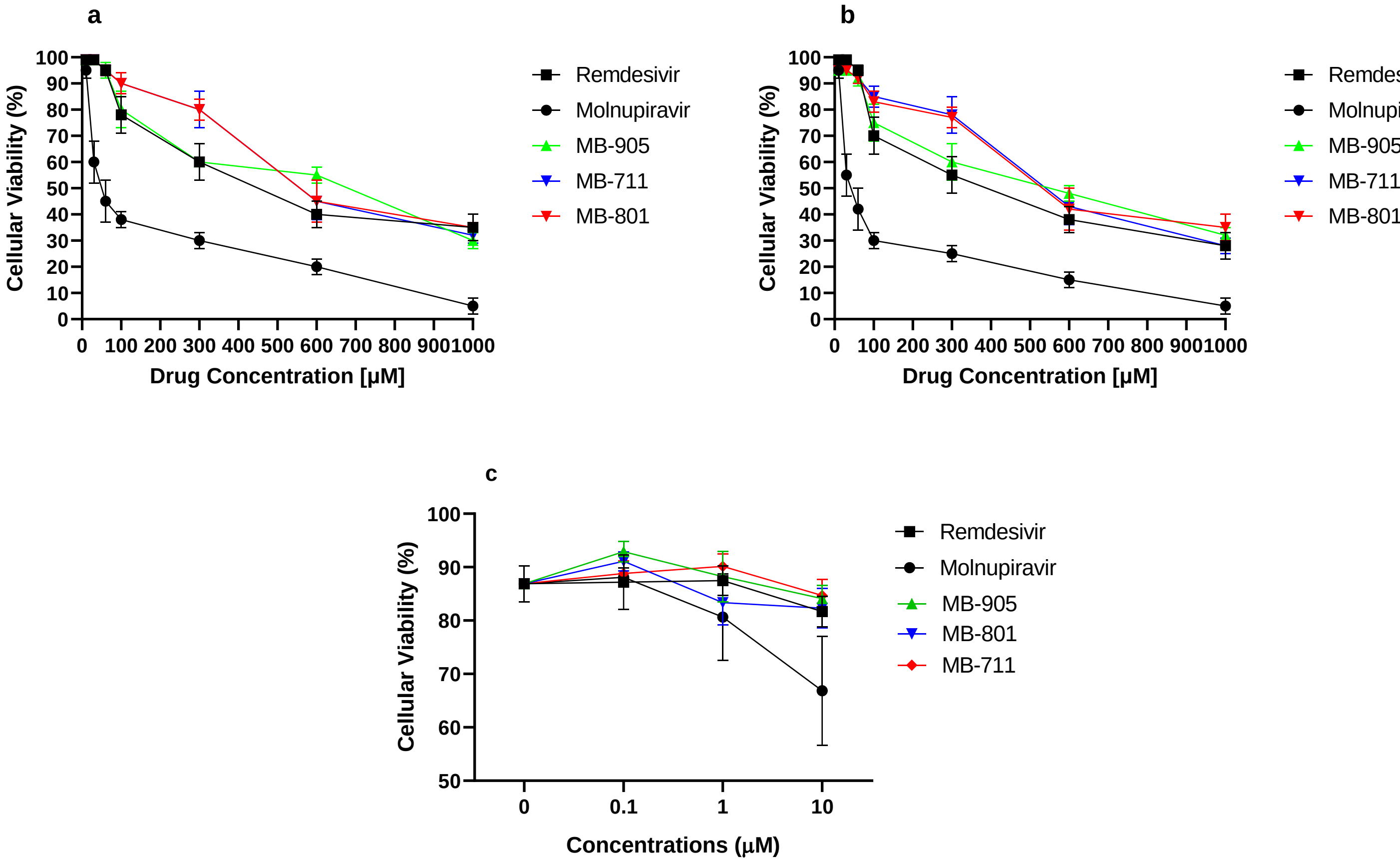

Supplementary Fig. 3. Citotoxicity of the compounds. Calu-3 cells at a density of 1.5 x 10^4 cells/well in 96-well plates were exposed to the indicated concentration of the compounds in DMEM containing 2% fetal bovine serum. After 24 h (**a**) or 48 h (**b**) of incubation, the cells were incubated with XTT/PMS to monitor for 4 h, and cellular viability was measured with a spectrophotometer at 492 nm and 620 nm (n=3 for panels **a** and **b**). Monocytes at a density of 1.5 x 10^4 in 48-well plates were incubated with the compounds in DMEM with 10% human sera (n=5) (**c**). After 24 h, the levels of LDH were measured in the supernatant of the cultures. The data represent the means ± SEMs of experiments with cells from at least three healthy donors of monocytes. MB-905, its corresponding ribonucleoside (MB-801) and monophosphoramidate

(MB-711) are displayed. Remdesivir (RDV) and molnupiravir (MK-4482) were used as positive controls.

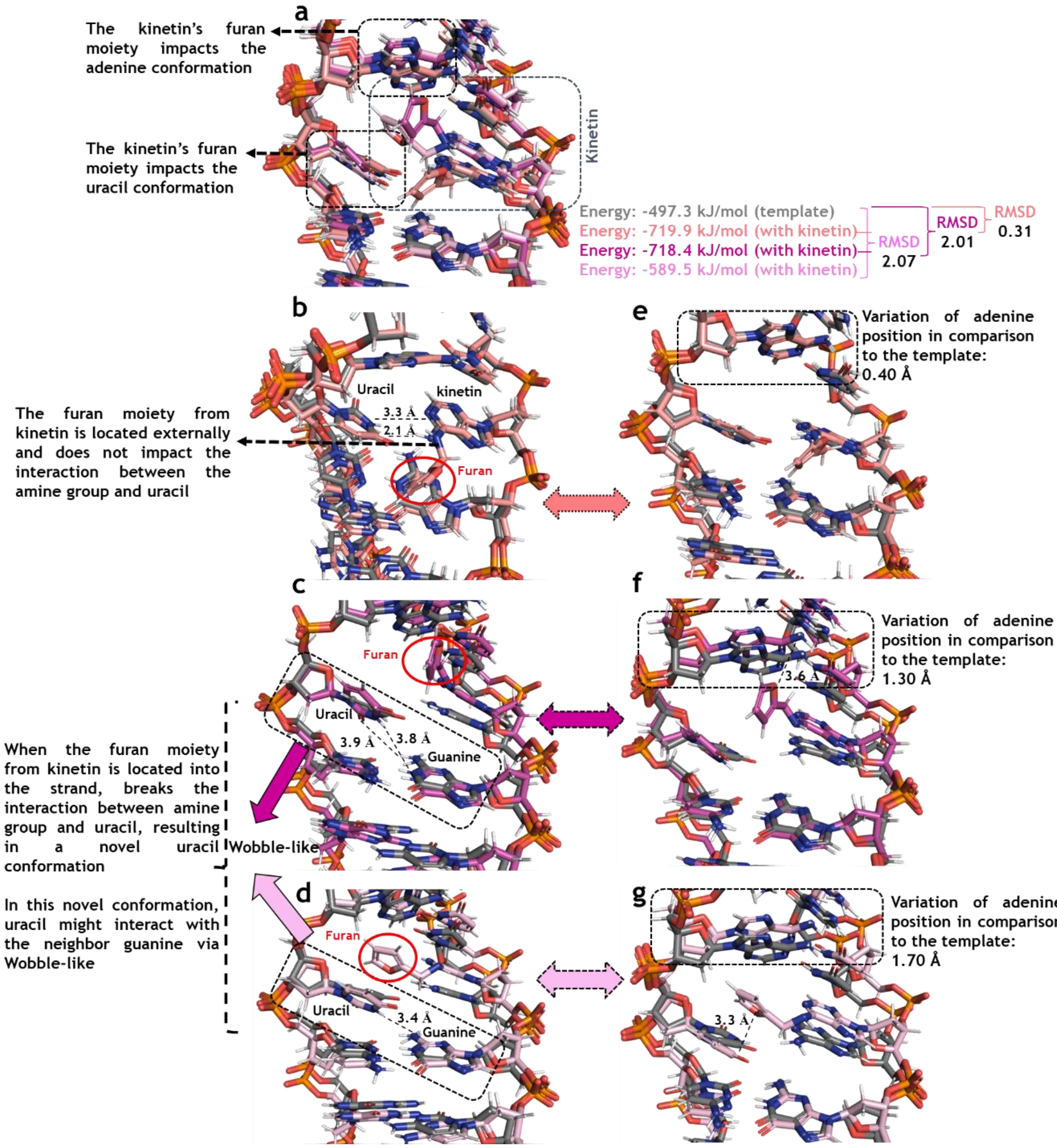

Supplementary Fig. 4. Molecular model of promiscuous pairing of tautomeric kinetin (MB-905). (**a**) Superposition of the three conformations (beige, magenta, and pink) in which furfuryl from kinetin affects the double-strand conformation (template in gray). (**b**, **c**, **d**) Molecular model in which furfuryl from kinetin affects the U conformation and interaction with the amine groups of kinetin and the neighbor G (resulting in a Wobble-like interaction). This model is in line with an error-prone mechanism. (**e**, **f**, **g**) Molecular model in which furfuryl from kinetin affects

neighbor An in the double-strand conformation. This model is in line with a possible steric hindrance of the RNA polymerase. Black dots indicate the interaction via hydrogen bonding, and RMSD means root-mean-square deviation. Element colors: hydrogen, oxygen, phosphorus, and nitrogen in white, red, orange, and blue, respectively.

Supplementary Table 2. Pharmacological parameters for MB905 and control RdRp inhibitors alone and in combination with SARS-CoV-2 nsp14 inhibitors in Calu-3 cells.

	EC50		EC90		EC99	
**Drug**	**mean**	**SEM**	**mean**	**SEM**	**mean**	**SEM**
Tenofovir	4.3	2.1	ND	ND	ND	ND
RDV	0.09	0.002	0.4	0.03	1.1	0.2
Favipiravir	7.8	1.2	ND	ND	ND	ND
MK-4482	0.8	0.03	7	0.4	9	0.7
**MB905**	0.3	0.02	8	1.2	ND	ND
DTG	5.3	1.2	ND	ND	ND	ND
RTG	4.8	1.4	ND	ND	ND	ND
Pibrentasvir	0.7	0.2	4.2	0.6	19	2
Ombitasvir	0.4	0.05	3.3	0.5	18	3
Daclatasvir	0.7	0.08	3.8	1.2	ND	ND
Tenofovir + DTG (5µM)	0.5	0.03	7	1.2	9.8	0.2
RDV+DTG (5µM)	0.09	0.004	0.4	0.03	0.9	0.2
Favipiravir + DTG (5µM)	0.15	0.07	8	1.3	9.8	0.2
MK-4482 + DTG (5µM)	0.03	0.004	8	1.2	9	0.7
**MB905 + DTG (5µM)**	0.06	0.004	5	0.9	8.7	0.5
Tenofovir + RTG (5µM)	0.4	0.02	8	1.5	9.5	0.1
RDV+RTG (5µM)	0.08	0.002	0.5	0.08	1.2	0.1
Favipiravir + RTG (5µM)	0.16	0.07	6	1.6	9.2	0.3
MK-4482 + RTG (5µM)	0.01	0.002	7	1.4	9.1	0.5
**MB905 + RTG (5µM)**	0.05	0.002	6	0.6	8.5	0.2
Tenofovir + Pibrentasvir (0.1µM)	0.5	0.05	8	1.5	ND	ND
RDV + Pibrentasvir (0.1µM)	0.008	0.0009	0.07	0.03	0.3	0.09
Favipiravir + Pibrentasvir (0.1µM)	0.5	0.03	8	0.5	ND	ND
MK-4482 + Pibrentasvir (0.1µM)	5.4	0.3	7	0.3	7.8	0.5
**MB905 + Pibrentasvir (0.1µM)**	6.4	0.9	8	0.4	ND	ND
Tenofovir + Ombitasvir (0,1µM)	0.8	0.07	7	1.6	8.9	0.4
RDV + Ombitasvir (0.1µM)	0.08	0.003	0.01	0.05	0.5	0.2
Favipiravir + Ombitasvir (0.1µM)	0.15	0.04	8	0.4	9.5	0.4
MK-4482 + Ombitasvir (0.1µM)	0.13	0.02	4	0.5	7.8	0.6

**MB905 + Ombitasvir (0.1µM)**	0.3	0.04	8	1.3	ND	ND
Tenofovir + Dacltasvir (0.5µM)	0.01	0.004	6	1.2	7.5	0.5
RDV + Dacltasvir (0.5µM)	0.008	0.0006	0.1	0.06	0.5	0.1
Favipiravir + Dacltasvir (0.5µM)	0.12	0.05	8	0.5	ND	ND
MK-4482 + Dacltasvir (0.5µM)	0.02	0.008	3	0.4	8.1	0.3
**MB905 + Dacltasvir (0.5µM)**	0.4	0.03	4	0.4	8.8	0.2

Supplementary Table 3. Mutagenicity results of MB-905 tested in the Ames test strains TA 97a, TA 98, TA100, TA102 and TA 1535. The test was performed in the absence and presence of the metabolic activation system (8% of S9 in the mixture with required cofactors).

Treatments	Concentration (µg/mL)	TA 97a		TA 98		TA 100		TA 102		TA 1535	
		-S9	+S9	-S9	+S9	-S9	+S9	-S9	+S9	-S9	+S9
MB-905	8	-	-	-	-	-	-	-	-	-	-
	40	-	-	-	-	-	-	-	-	-	-
	200	-	-	-	-	-	-	-	-	-	-
	1,000	-	-	-	-	-	-	-	-	-	-
	5,000	-	-	-	-	-	-	-	-	-	-
Positive Control	#	+	+	+	+	+	+	+	+	+	+

(-S9) = absence of the metabolic activation system; (+S9) = presence of the metabolic activation system; (-) = negative test; (+) = positive test. # Positive controls = 4-nitroquinoline-N-oxide (4NQO) 0.5 µg/plate: TA97a, TA98 and TA102 (-S9); sodium azide (AZS) 1.5 µg/plate: TA100 and TA 1535 (-S9); 2-aminofluorene (2-AF) 50 µg/plate: TA97a, TA98 and TA100 (+S9); 2-aminoanthracene (2-AA): 2.5 and 5 µg/plate: TA 1535 and TA102, respectively (+S9). Each group represents triplicate experiments.

Supplementary Table 4. Incidence of micronucleated polychromatic erythrocytes (MNPCE) and the *ratio* of polychromatic erythrocytes (*PCE*) to normochromatic erythrocytes in mice treated with MB-905.

Group	Dose (mg/Kg)	Route	MNPCE/4,000 PCE (Mean ± S.D.)	P Value	Ratio PCE/NCE (Mean ± S.D.)
**Negative Control**	0	p.o.	10.10 ± 4.89	-	1.33 ± 0.18
**MB-905**	32	p.o.	8.50 ± 4.50	0.0400	1.26 ± 0.20
	125	p.o.	8.50 ± 3.81	> 0.9999	1.43 ± 0.24
	500	p.o.	9.00 ± 2.45	> 0.9999	1.26 ± 0.12
**Positive Control**	25	i.p.	16.20 ± 6.03*	> 0.9999	1.44 ± 0.29

PCE = polychromatic erythrocytes; NCE = normochromatic erythrocytes; Ratio PCE/NCE means the cytotoxicity of compounds. MNPCE = micronucleated polychromatic erythrocytes evaluated in 4000 cells; *p<0.05, significantly different from the negative control by Kruskal-Wallis test. p.o. = *per os*; i.p. = intraperitoneal. Negative control: 5% Tween 80 and 95% PEG 400 (polyethylene glycol 400), Positive Control: Cyclophosphamide. Data were expressed as mean ± SD (standard deviation). (n = 10).

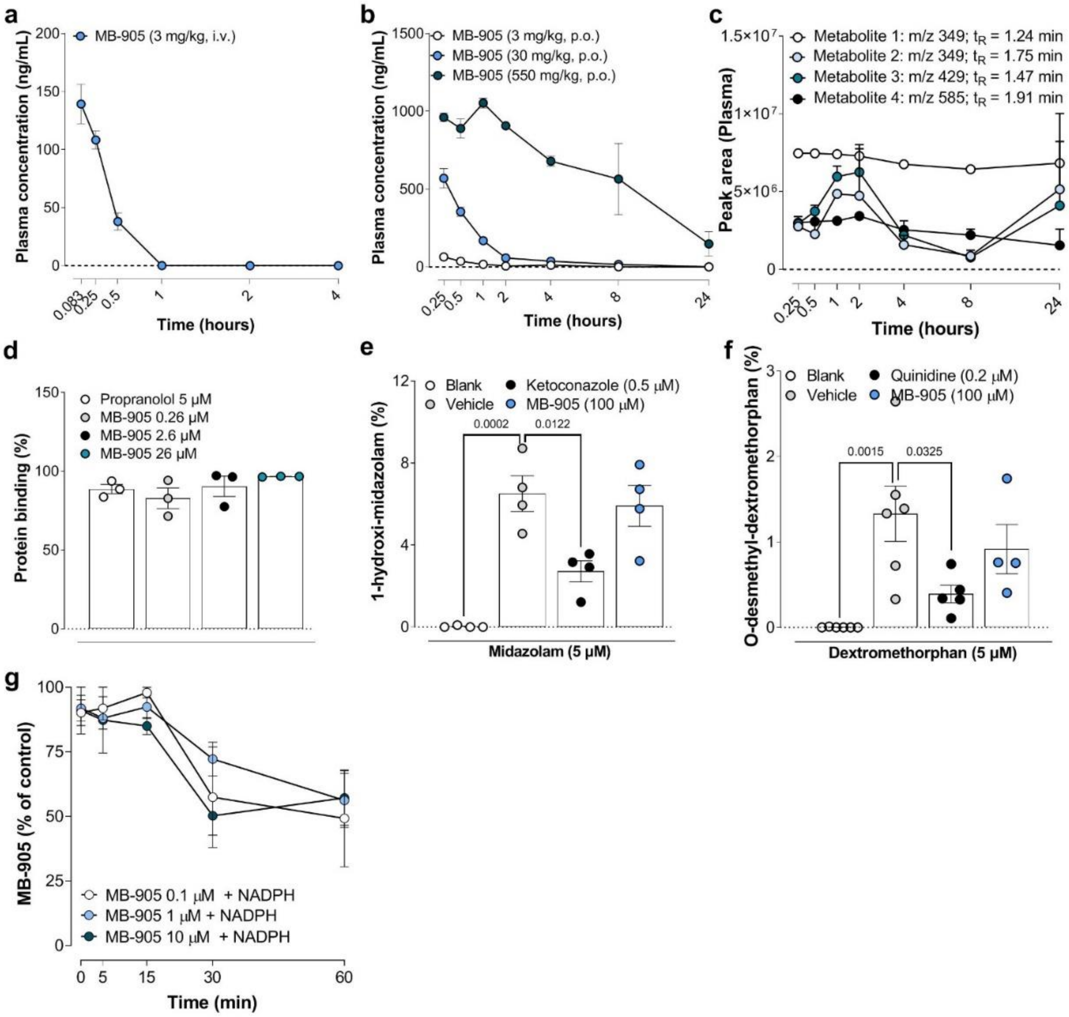

Supplementary Figure 5. Pharmacokinetics in mice, protein binding, CYP inhibition and metabolites. a, Single intravenous dose (3 mg/kg bodyweight) pharmacokinetics properties of MB-905 in mouse plasma (n = 6); **b**, Single oral doses (3, 30 and 550 mg/kg bodyweight) pharmacokinetics properties of MB-905 in mouse plasma (n = 2; n = 6; and n = 3, respectively); **c,** Plasma putative metabolites after treatment with MB-905 (550 mg/kg, p.o.) (n=3); **d,** Evaluation of *in vitro* protein binding percentage of MB-905 (0.26 – 26 µM) in mouse plasma (n = 3); **e,** MB-905 (100 µM) or ketoconazole (0.5 µM- an inhibitor of CYP3A4) were incubated with recombinant human CYP3A4 and midazolam and evaluated the production of 1-hydroxy-midazolam (n = 3); **f,** MB-905 or quinidine (0.2 µM- an inhibitor of CYP2D6) were incubated

with recombinant human CYP2D6 and dextromethorphan and evaluated the production of O-demethyl-dextromethorphan (n = 3); **g,** MB-905 (0.1, 1 and 10 μM) was incubated with human liver microsome (HLM) in the presence and in the absence of NADPH following analysis of MB-905 concentrations at 0, 5, 15, 30 and 60 minutes after NADPH (n = 3; n = 2; and n = 2, respectively). Data are expressed as the mean ± SEM (standard error of the mean). **d-f:** One-way ANOVA followed by Tukey's test was performed. Noncompartmental data analysis was performed using Phoenix WinNonlin®.

Supplementary Table 5. Pharmacokinetic parameters for MB-905 in mice.

Compound	MB-905		
Dose and Route	3 mg/kg (i.v.)	30 mg/kg (p.o.)	550 mg/kg (p.o.)
C_{max} (ng/mL)	155.16	569.97	1053.37
T_{max} (h)	0.083	0.083	0.5
$T_{1/2}$ (h)	0.22	1.11	2.72
CL (mL/min/kg)	918.38	1060.15	1843.18
Vz (L/kg)	19.04	102.21	434.2
AUC_{last} (h*ng/mL)	55.48	355.63	4392.27
AUC_{all} (h*ng/mL)	66.41	355.63	4392.27
Ke (1/h)	3.09	0.62	0.25
F(%)	100%	53.50%	36.10%

C_{max}: Peak concentration; T_{max}: Time to reach C_{max}; $T_{1/2}$: Half-life; CL: Clearance; V_z: Volume of distribution; AUC_{last}: Area under the curve (last); AUC_{all} area under the curve (all); K_e: elimination rate constant; F: bioavailability. Noncompartmental data analysis was performed using Phoenix WinNonlin®. Data represent the mean values of 3-6 animals per group.

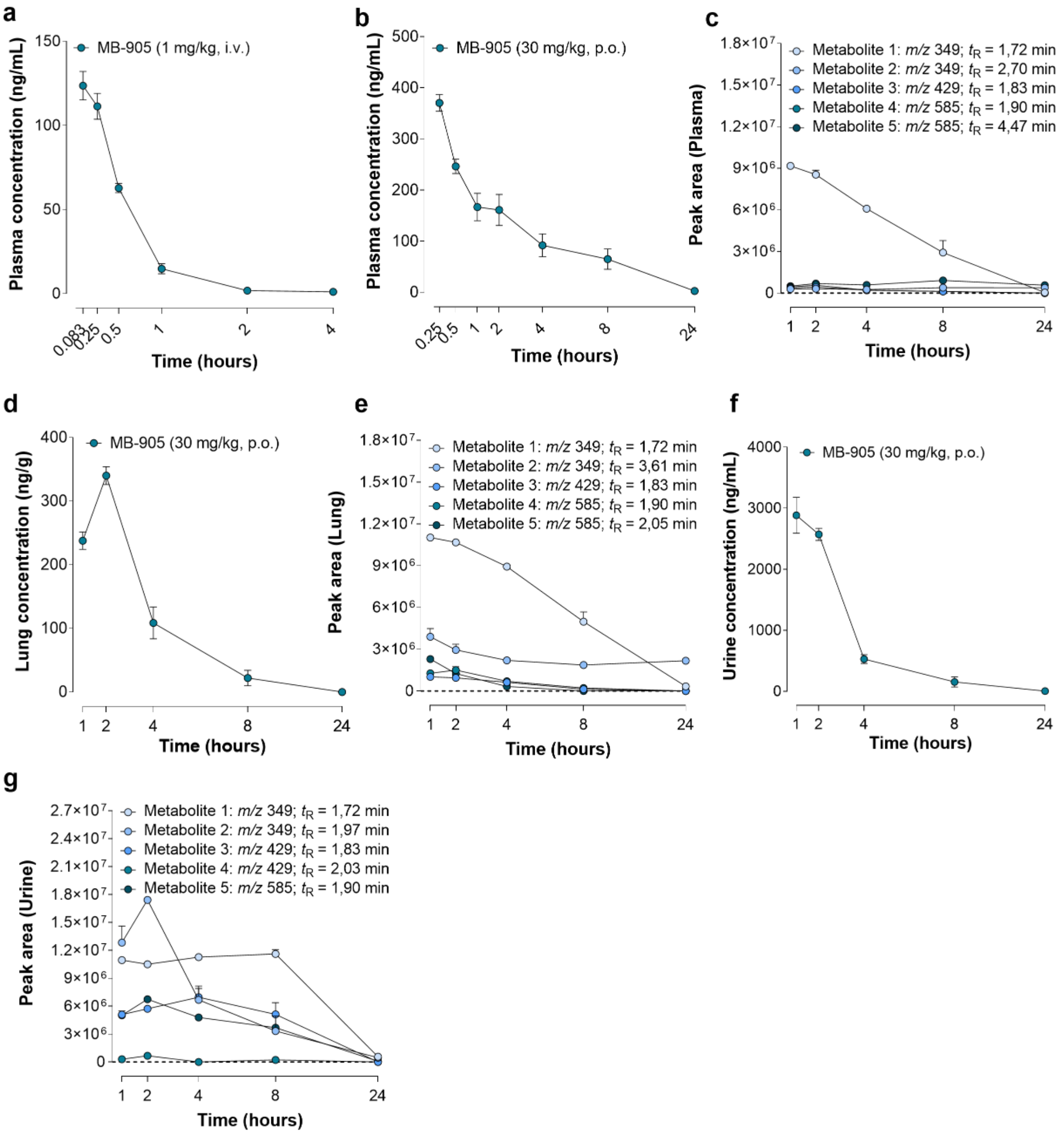

Supplementary Fig. 6. Pharmacokinetic and putative metabolites of MB-905 in rats. a, Single intravenous dose (1 mg/kg bodyweight) pharmacokinetics properties of MB-905 in rat plasma (n = 6); **b**, Single oral dose (30 mg/kg bodyweight) pharmacokinetics properties of MB-905 in rat plasma (n = 5); **c**, Plasma metabolites (5 metabolites) after treatment with MB-905 (30 mg/kg, p.o.) (n=5) **d,** Single oral dose (30 mg/kg bodyweight) pharmacokinetics properties of MB-905 in lung plasma (n = 3). **e,** Lung metabolites (5 metabolites) after treatment with MB-905 (30 mg/kg, p.o.) (n=3) **f,** Single oral dose (30 mg/kg bodyweight) pharmacokinetics properties of

MB-905 in rat urine (n = 3); **g,** Urine metabolites (5 metabolites) after treatment with MB-905 (30 mg/kg, p.o.) (n = 3). Data are expressed as the mean ± SEM (standard error of the mean). Noncompartmental data analysis was performed using Phoenix WinNonlin®.

Supplementary Table 6. Pharmacokinetic parameters for MB-905 in rats.

**Compound**	**MB-905**	
**Dose and Route**	1 mg/kg (i.v.)	30 mg/kg (p.o.)
**C_{max} (ng/mL)**	123.61	370.47
**Tmax (h)**	0.083	0.25
**$T_{1/2}$ (h)**	0.56	3.81
**CL (mL/min/kg)**	213.63	330.53
**Vz (L/kg)**	10.4	109.18
**AUC_{last} (h*ng/mL)**	77.12	1498.08
**AUC_{all} (h*ng/mL)**	77.12	1498.08
**Ke (1/h)**	1.23	0.18
**F(%)**	100%	64.70%

C_{max}: Peak concentration; T_{max}: Time to reach C_{max}; $T_{1/2}$: Half-life; CL: Clearance; V_z: Volume of distribution; AUC_{last}: Area under the curve (last); AUC_{all}: area under the curve (all); K_e: elimination rate constant; F: bioavailability. Noncompartmental data analysis was performed using Phoenix WinNonlin®. Data represent the mean values of 5-6 animals per group.

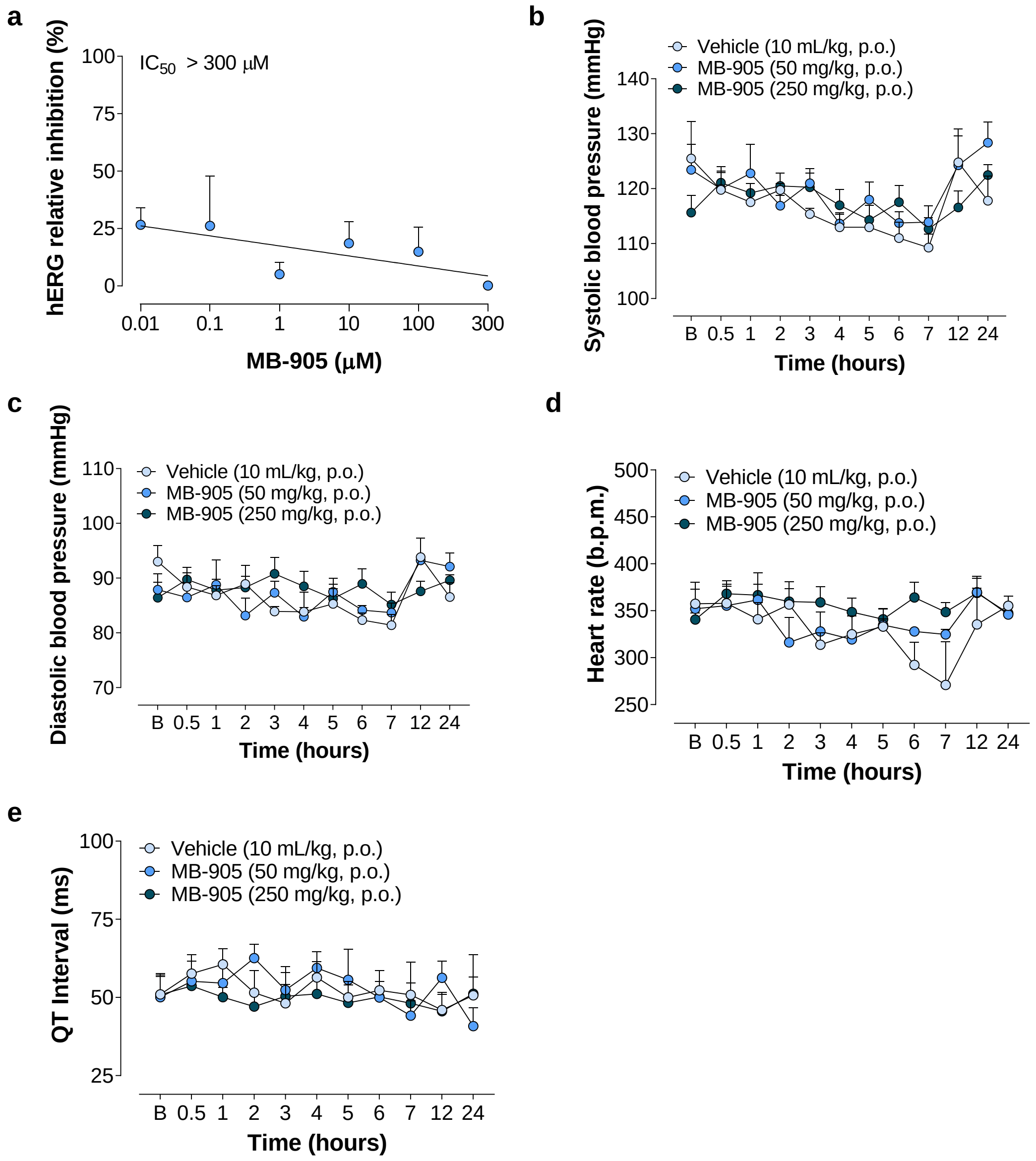

Supplementary Fig. 7. hERG channel assay *in vitro* and cardiovascular safety pharmacology in *vivo*. a, Concentration–response curve of MB-905 in the hERG channel inhibition assay (% of hERG relative inhibition). Data are expressed as the mean ± SEM (n=3) through nonlinear regression; **b**, systolic blood pressure in millimeters of mercury (mmHg); **c**, diastolic blood pressure in mmHg; **d**, heart rate expressed as beats per minute (bpm); **e**, QT interval in milliseconds (ms). **b-e:** values were obtained before (baseline, B)

and after oral treatment with vehicle (5 ml/kg, p.o., n = 4), MB-905 (50 mg/kg, p.o., n = 5) or MB-905 (250 mg/kg, p.o., n = 6) for 7 consecutive days. Data are expressed as the mean ± SEM. Statistical analyses were performed using a mixed effects model followed by Dunnett's test. in rats treated with vehicle (5 ml/kg, p.o.) or MB-905 (50 or 250 mg/kg, p.o.) administered orally once a day for 7 consecutive days. Data are expressed as the mean ± SEM (n= 4-6). **c-f**: Statistical analyses were performed using a mixed effects model followed by Dunnett's test.

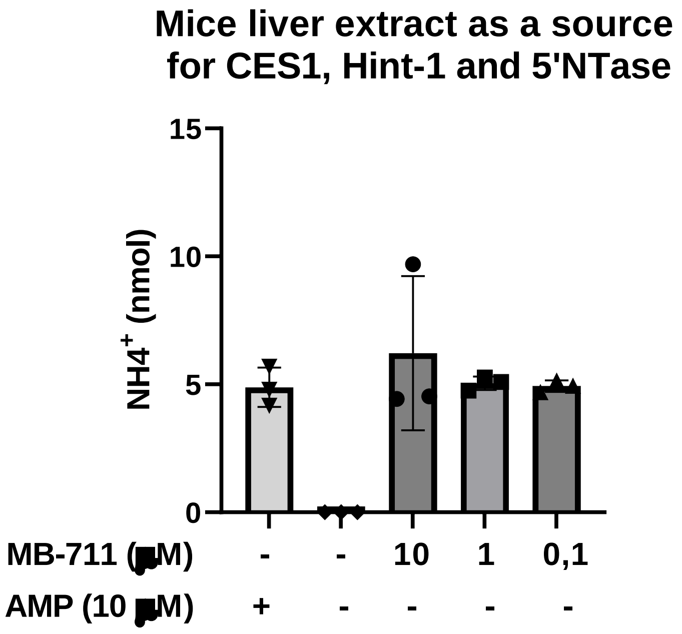

Supplementary Fig. 8. Kinetin-ribose-5'-monophosphate as a substrate for 5'-nucleotidase. Liver extracts from untreated Swiss webster mice, 20 weeks old, were incubated with MB-711 (Kinetin-ribose-5'-monophosphoramidate) or AMP as a substrate for a commercial reaction to detect 5'-nucleidase activity. Liver extract enzymes cathepsin A or carboxylesterase 1 and histidine triad nucleotide-binding protein 1 (HINT1) released nucleotide monophosphate from MB-711 to be further used by 5'-nucleotidase (#ab235945 from www.abcam.com). Each column represents the means ± SEMs of three independent experiments.

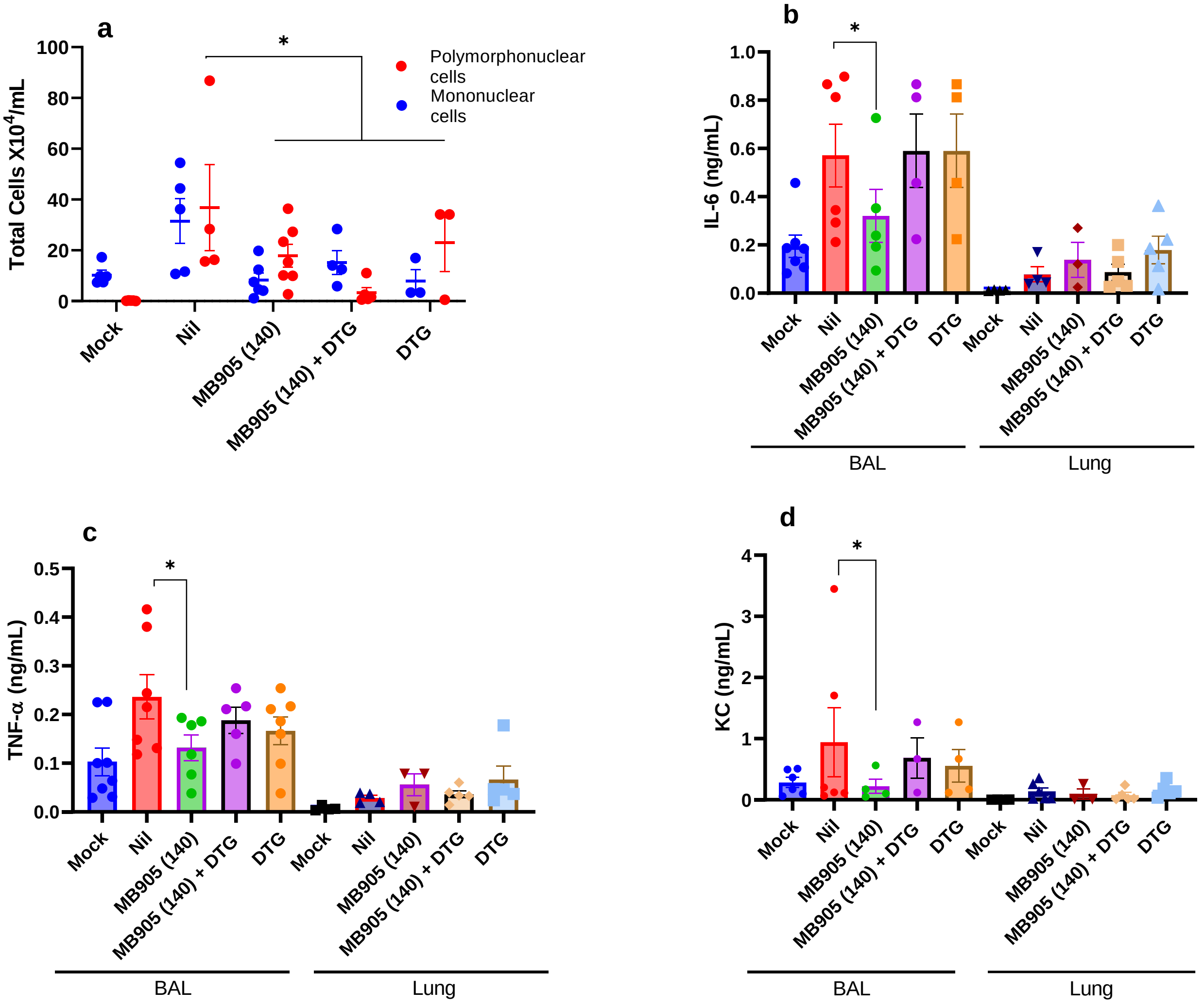

Supplementary Fig. 9. MB-905 attenuates SARS-CoV-2-induced inflammation in transgenic K18 mice. Transgenic mice expressing the hACE2 receptor to SARS-CoV-2 entry at the age of 10-12 weeks were infected with 105 PFU intranasally. After 12-18 h, the treatments were performed and maintained daily. Polymorphonuclear and mononuclear cells were measured in bronchoalveolar lavage (BAL) (n=3-7) (A). ELISAs to quantify IL-6 (n=3-7) (B), TNF-α (n=

3-8) (C) and (n=3-6) KC (D) were performed in the BAL fluid and lungs of the animals. Data are presented as mean values ± SEM and *$P<0.05$ by ordinary one-way ANOVA analysis.

Supplementary Chemical information

***Kinetin, MB-905* (A)**[1]

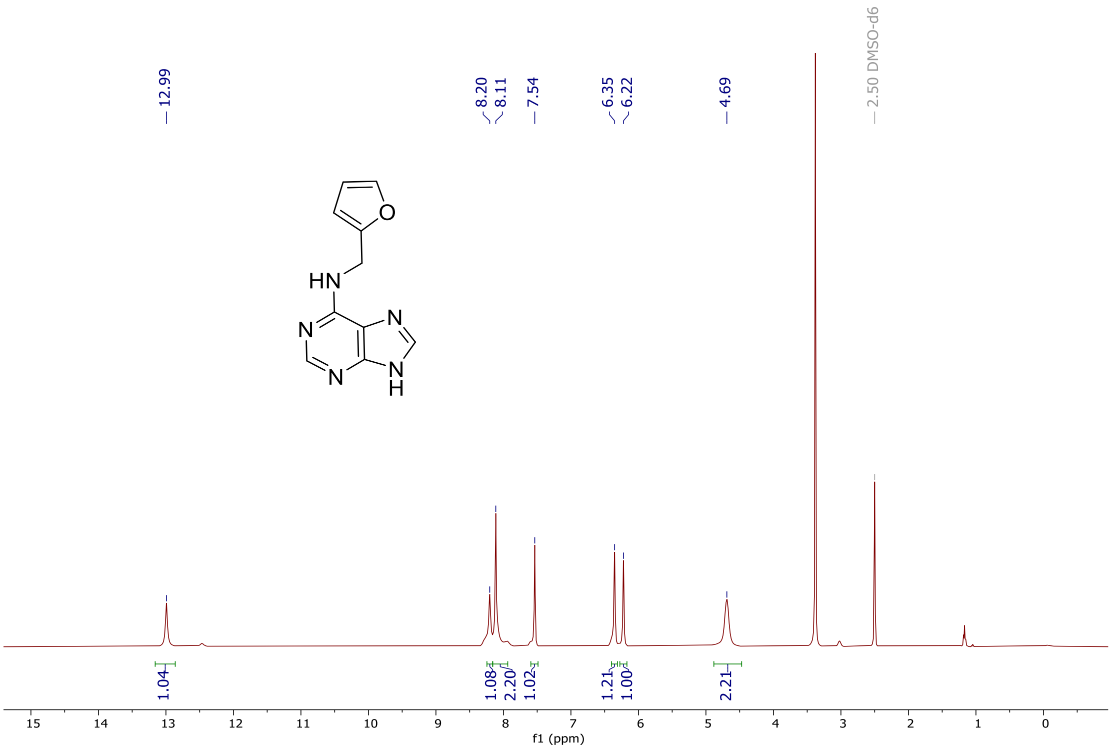

^{1}H NMR (500 MHz, $CDCl_3$) of compound MB-905 (**A**)

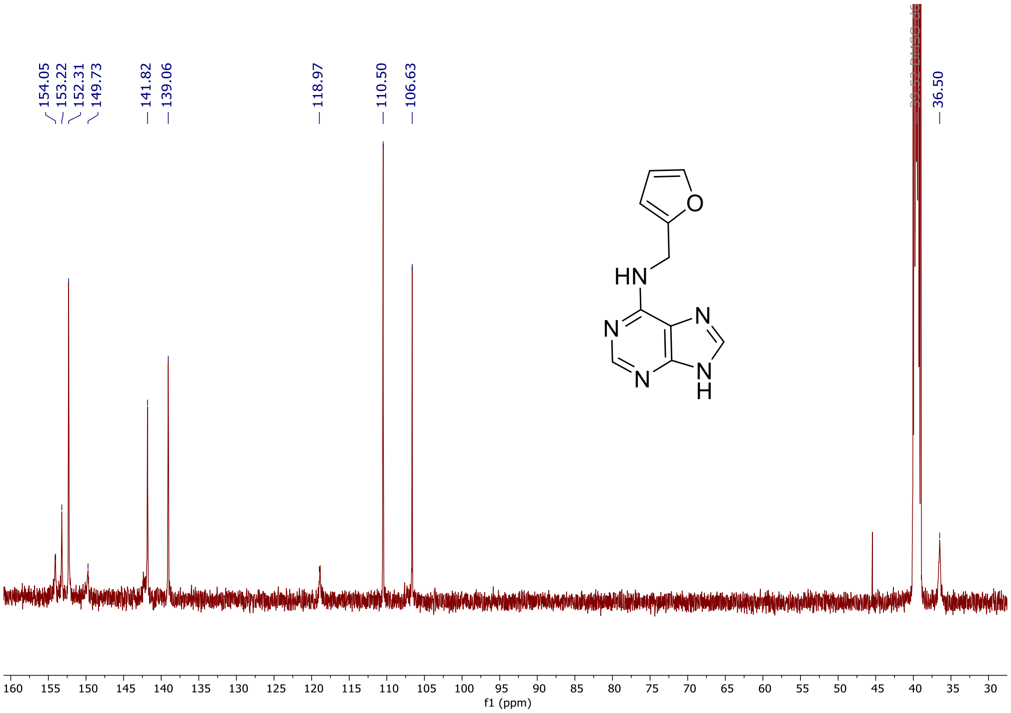

^{13}C NMR (126 MHz, $CDCl_3$) of compound MB-905 (**A**)

D:\Dados\Cinetina\Desenvolvimento\Métodos\Cinetina_XTerra RP18_CRM 007.lcm
D:\Dados\Cinetina\Desenvolvimento\2021\09-Set\Cinetina_XTerra RP18_TFA_CRM 007_080921_2.lcd
Acquired: 08/09/2021 09:50:27 Date Process: 08/09/2021 10:40:33
Sample ID: Cinetina
Data Description: CN.01/21S1
Diluição: 25,1mg + 20mL NaOH 0,1N/50mL
Fase Móvel: Iso 10% Fase B [MeOH:ACN (1:1)] : 90% Fase A [3,4g/L KH2PO4 pH2,54 (H3PO4)]
Temp. Forno: 30°C
Coluna: X Terra RP18 250 x 4,6mm 5um (02593832313854)
Fluxo: 1,0mL/min Pressão Inicial: 107kgf/cm2
Vol. de injeção: 20uL
User Name: Pesquisa e Desenvolvimento

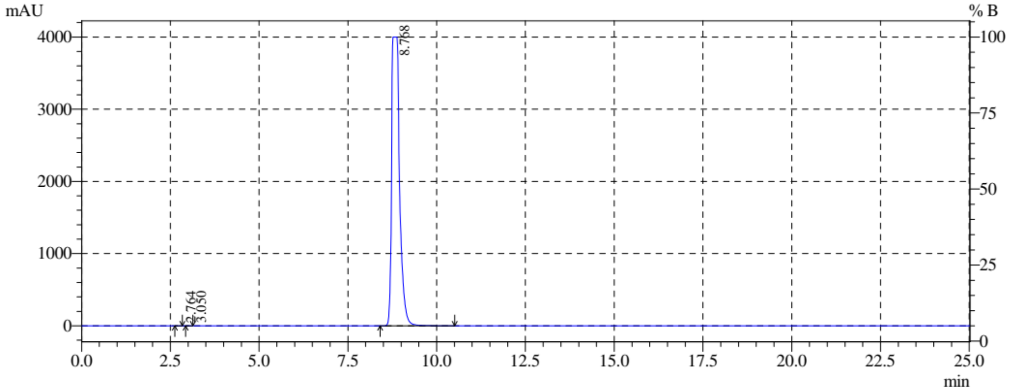

1 PDA Multi 1 / 210nm 4nm

PDA Ch1 210nm 4nm

Peak#	Ret. Time	Area	Area %	Theoretical Plate#	Resolution	Tailing Factor	Lambda max
1	2.764	13410	0.021	3034.506	0.000	0.000	663/674/772/583/546
2	3.050	8391	0.013	4347.799	1.480	0.000	795/771/743/583/549
3	8.768	62444202	99.965	11616.785	22.406	2.229	205/272/657/674/631
Total		62466002	100.000				

HPLC of compound MB-905 (**A**)

6-furfurylamino-9-(tetrahydropyran-2-yl)-9H-purine, MB-906 (**B**)[2]

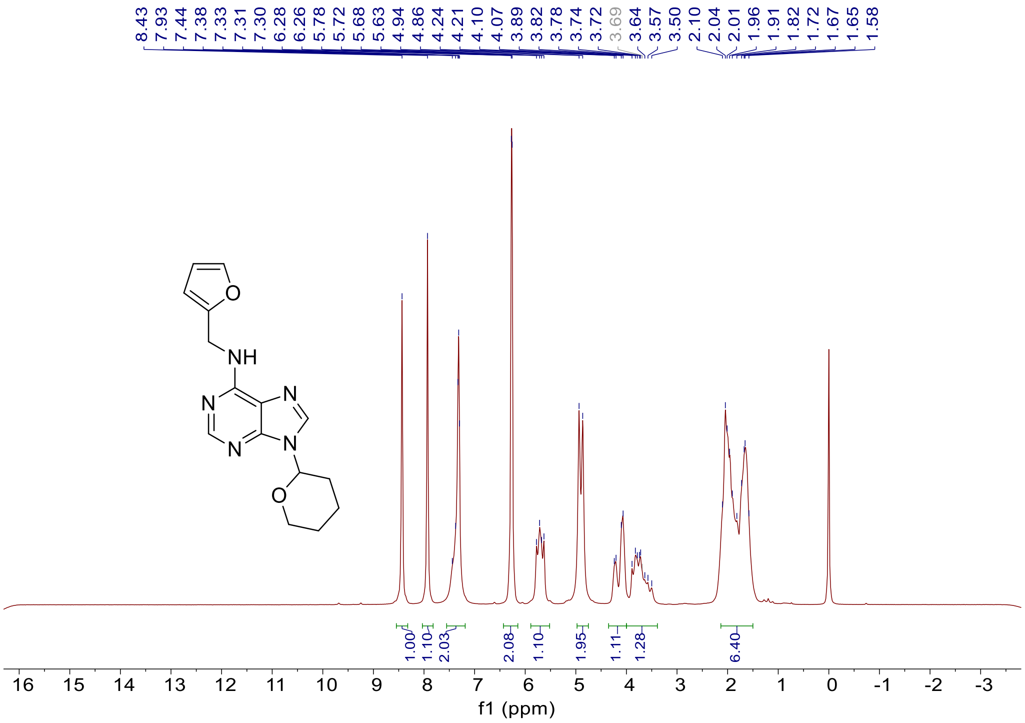

¹H NMR (80 MHz, $CDCl_3$) of compound MB-906 (**B**)

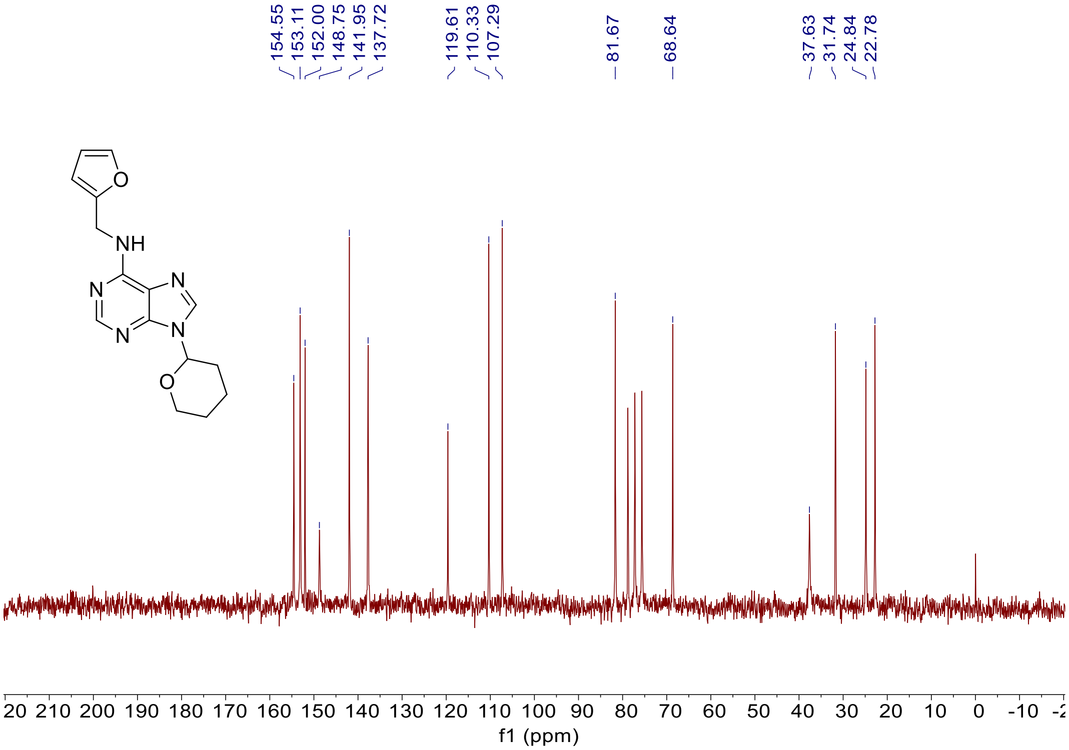

^{13}C NMR (20 MHz, $CDCl_3$) of compound MB-906 (**B**)

Z:\HPLC\P&D\Tritil-Zeatina\Tritil-Zeatina_XTerra RP18_TFA_CRM 007.lcm
Z:\HPLC\P&D\Tritil-Zeatina\2021\11-Nov\Tritil-Zeatina_XTerra RP18_TFA_CRM 007_291121_14.lcd
Acquired: 29/11/2021 20:53:31 Date Process: 29/11/2021 21:23:41
Sample ID: Amostra
Data Description: MB-CN-THP.001.2016
Diluição: Am + ACN/10mL 95% H2O/ACN
Fase Móvel: Grad 5-100% Fase B [90% ACN/10% H2O (0,1% TFA)] : Fase A [90% H2O (0,1% TFA)/10% ACN]
Temp. Forno: 30°C
Coluna: X Terra RP18 250 x 4,6mm 5um (02593832313854)
Fluxo: 1,2mL/min Pressão Inicial: 129kgf/cm2
Vol. de injeção: 10uL
User Name: lara

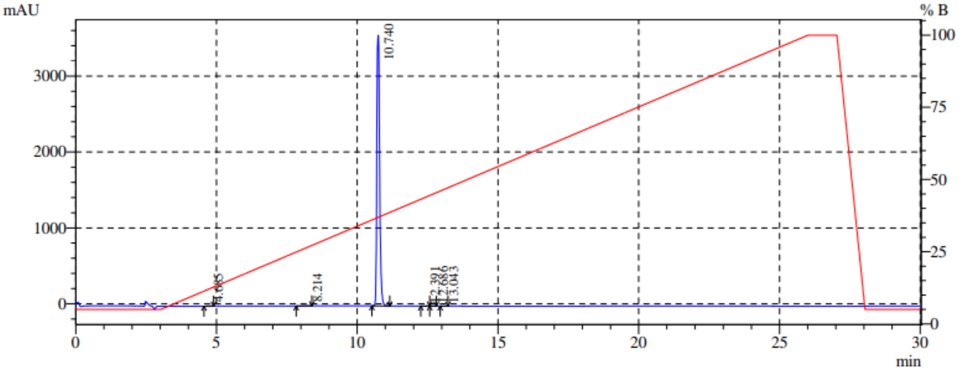

1 PDA Multi 1 / 210nm 4nm

PDA Ch1 210nm 4nm

Peak#	Ret. Time	Area	Area %	Theoretical Plate#	Resolution	Tailing Factor	Lambda max
1	4.685	29547	0.120	6261.508	0.000	1.346	275/774/704/688/485
2	8.214	11846	0.048	17558.058	14.556	0.926	642/272/704/774/485
3	10.740	24470202	99.708	56232.770	11.775	1.307	213/263/667/641/485
4	12.391	12294	0.050	69530.741	8.945	1.392	667/270/485/704/569
5	12.686	5397	0.022	97718.246	1.686	1.182	668/641/270/485/711
6	13.043	12457	0.051	93410.744	2.141	1.135	642/274/485/774/687
Total		24541742	100.000				

HPLC of compound MB-906 (**B**)

***Phenyl (Isopropoxy-L-alaninyl) Kinetin Riboside Phosphoramidate, MB-711* (C)**[3]

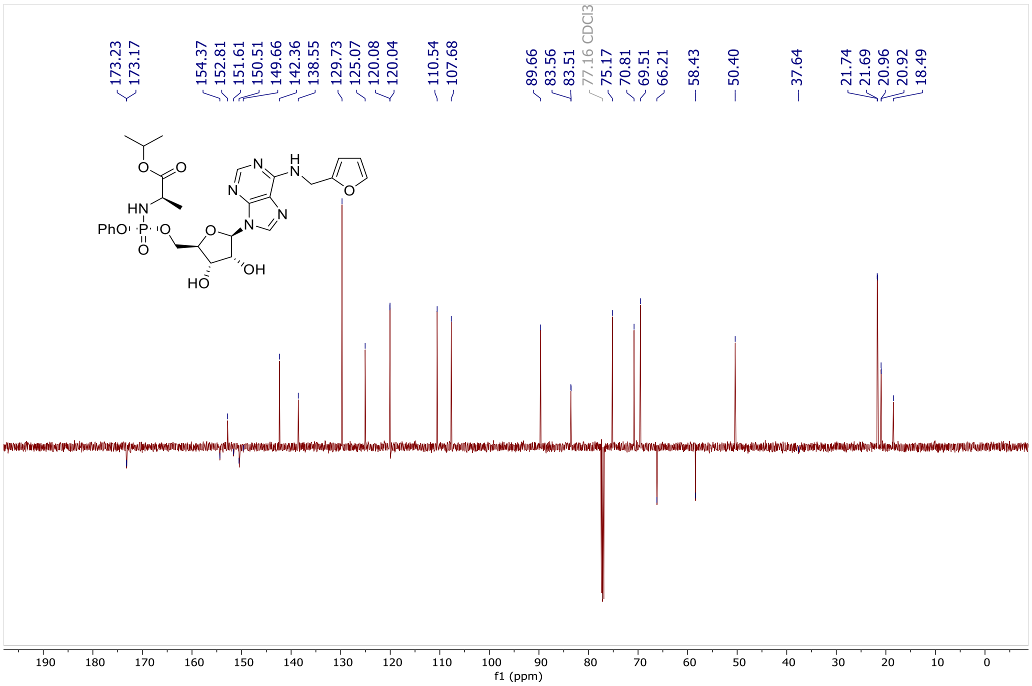

^{1}H NMR (500 MHz, $CDCl_3$) of compound MB-711 (**C**)

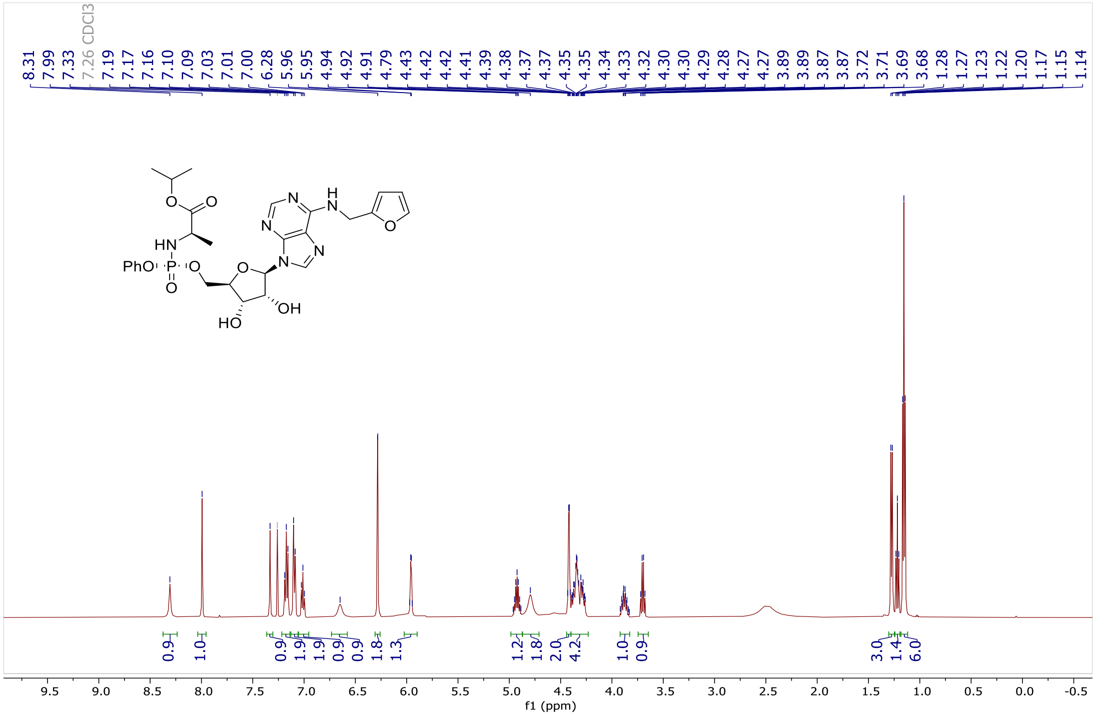

^{13}C (Dept-135) NMR (126 MHz, $CDCl_3$) of compound MB-711 (**C**)

Z:\HPLC\P&D\Tritil-Zeatina\Tritil-Zeatina_XTerra RP18_TFA_CRM 007.lcm
Z:\HPLC\P&D\Tritil-Zeatina\2021\11-Nov\Tritil-Zeatina_XTerra RP18_TFA_CRM 007_291121_4.lcd
Acquired: 29/11/2021 15:47:33 Date Process: 29/11/2021 16:17:45
Sample ID: Amostra
Data Description: MB-711
Diluição: Am + ACN/10mL 95% H2O/ACN
Fase Móvel: Grad 5-100% Fase B [90% ACN/10% H2O (0,1% TFA)] : Fase A [90% H2O (0,1% TFA)/10% ACN]
Temp. Forno: 30°C
Coluna: X Terra RP18 250 x 4,6mm 5um (02593832313854)
Fluxo: 1,2mL/min Pressão Inicial: 129kgf/cm2
Vol. de injeção: 10uL
User Name: lara

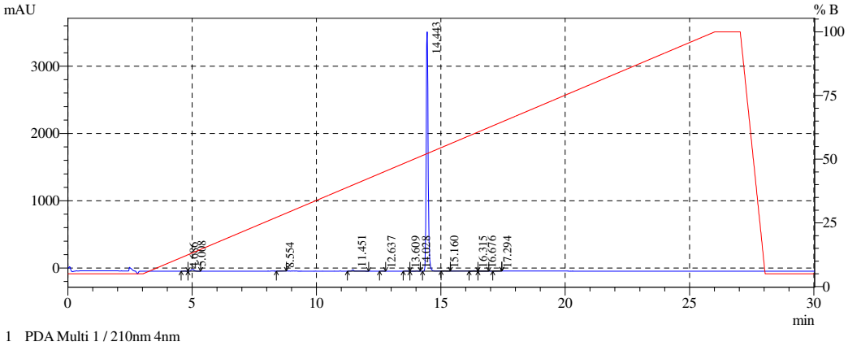

1 PDA Multi 1 / 210nm 4nm

PDA Ch1 210nm 4nm

Peak#	Ret. Time	Area	Area %	Theoretical Plate#	Resolution	Tailing Factor	Lambda max
1	4.686	15813	0.080	7337.973	0.000	0.000	663/644/273/775/539
2	5.008	223687	1.127	6859.670	1.399	1.181	267/667/643/694/765
3	8.554	11101	0.056	18907.199	14.452	1.421	668/643/485/270/711
4	11.451	100112	0.504	81736.627	14.163	1.169	258/667/641/774/330
5	12.637	11079	0.056	102416.749	7.453	1.024	667/269/630/765/330
6	13.609	14899	0.075	95189.445	5.811	1.039	662/267/485/772/330
7	14.028	7662	0.039	86053.100	2.282	0.000	644/287/485/774/689
8	14.443	19362954	97.575	140090.443	2.399	1.278	212/264/662/485/688
9	15.160	26115	0.132	124928.393	4.402	1.236	667/269/694/773/782
10	16.315	36814	0.186	143403.289	6.717	1.305	667/643/269/694/774
11	16.676	19158	0.097	112904.350	1.947	0.000	667/641/273/485/774
12	17.294	14789	0.075	46659.238	2.383	0.865	662/273/705/774/741
Total		19844181	100.000				

HPLC of compound MB-711 (**C**)

Kinetin riboside, MB-801 (**D**)[3]

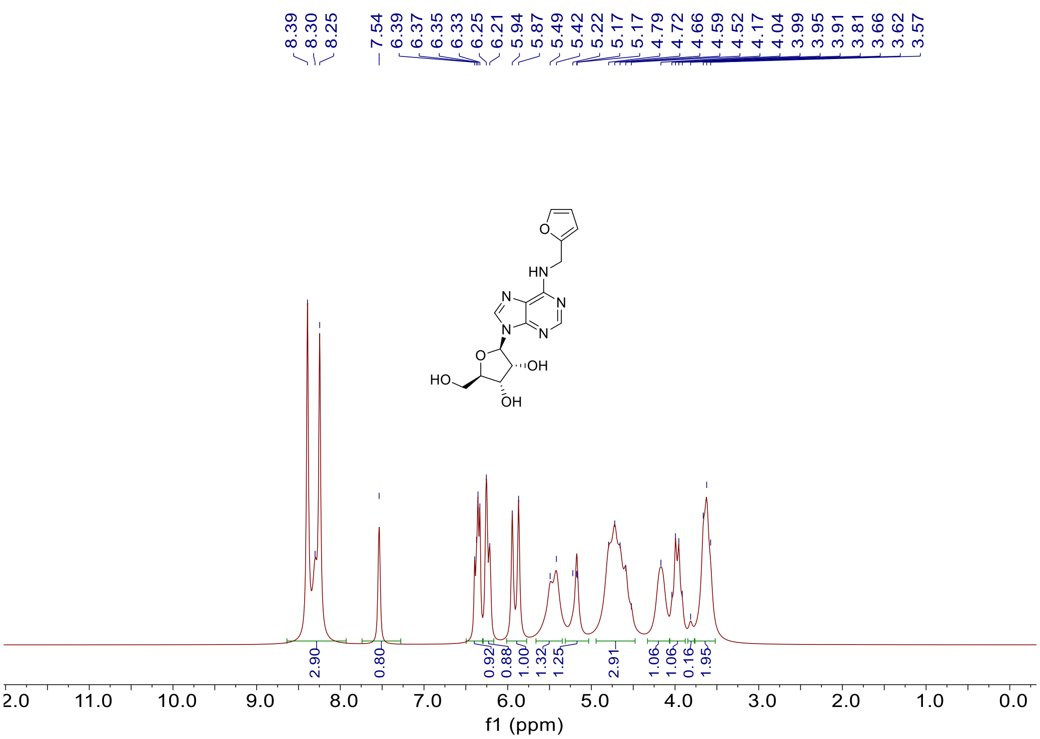

^{1}H NMR (80 MHz, $CDCl_3$) of compound MB-801 (**D**)

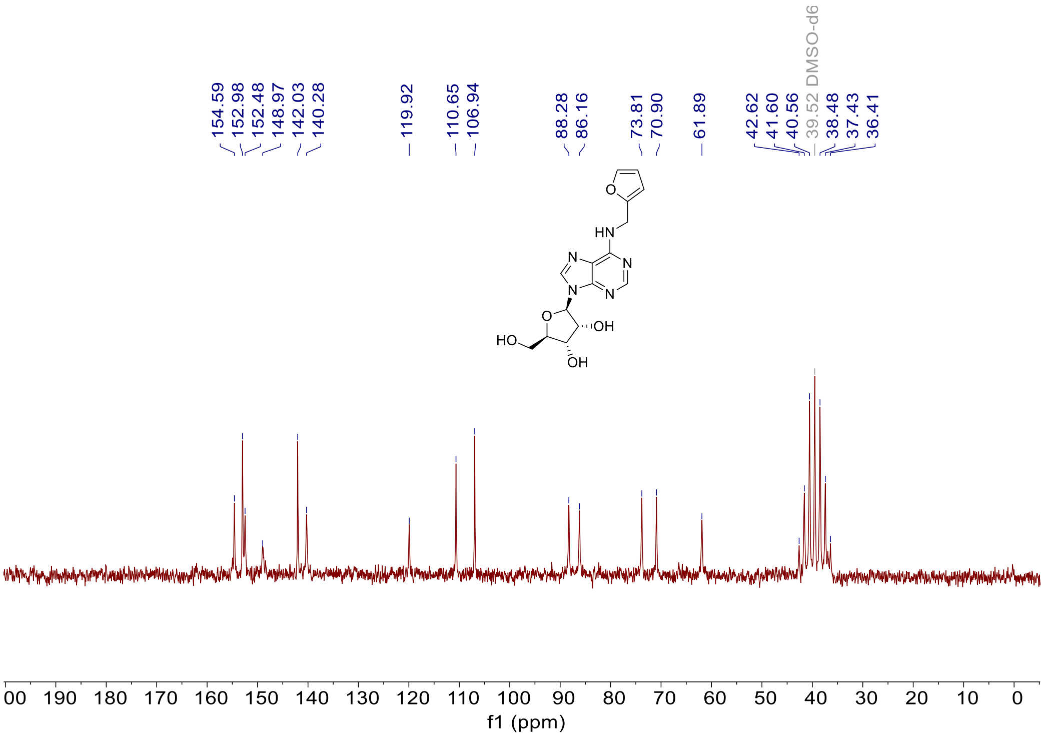

^{13}C NMR (20 MHz, DMSO-d_6) of compound MB-801 (**D**)

Acquired: 29/11/2021 21:24:06 Date Process: 29/11/2021 21:54:17
Sample ID: Amostra
Data Description: MB-801
Diluição: Am + ACN/10mL 95% H2O/ACN
Fase Móvel: Grad 5-100% Fase B [90% ACN/10% H2O (0,1% TFA)] : Fase A [90% H2O (0,1% TFA)/10% ACN]
Temp. Forno: 30°C
Coluna: X Terra RP18 250 x 4,6mm 5um (02593832313854)
Fluxo: 1,2mL/min Pressão Inicial: 129kgf/cm2
Vol. de injeção: 10uL
User Name: lara

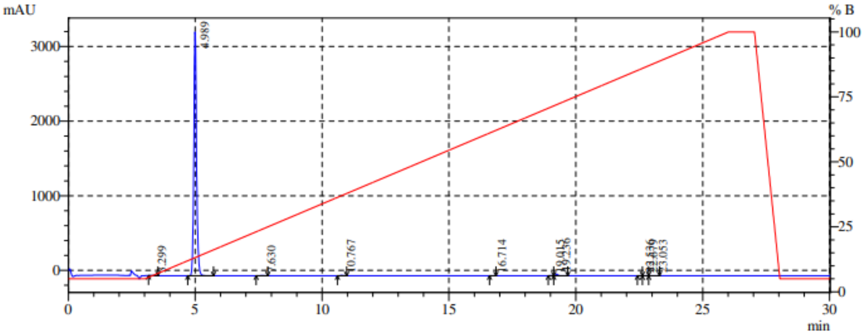

1 PDA Multi 1 / 210nm 4nm

PDA Ch1 210nm 4nm

Peak#	Ret. Time	Area	Area %	Theoretical Plate#	Resolution	Tailing Factor	Lambda max
1	3.299	10594	0.043	2593.918	0.000	1.069	667/643/485/271/711
2	4.989	24297617	98.914	9442.944	7.280	1.207	211/265/642/485/796
3	7.630	28425	0.116	12932.222	11.149	1.023	642/676/271/485/694
4	10.767	37750	0.154	53652.777	13.809	1.189	667/647/270/485/703
5	16.714	5987	0.024	146063.498	32.960	1.273	643/485/703/716/790
6	19.015	22043	0.090	189326.215	13.156	0.000	667/642/485/704/273
7	19.236	144458	0.588	202395.122	1.282	1.269	667/265/642/485/703
8	22.536	5107	0.021	138559.379	15.970	0.000	642/716/689/773/274
9	22.679	5936	0.024	170629.371	0.622	0.000	485/704/273/783/592
10	23.053	6492	0.026	157005.052	1.651	1.309	668/647/485/704/771
Total		24564409	100.000				

HPLC of compound MB-801 (**D**)
