# Supplementary material for: Preclinical development of kinetin as a safe error-prone SARS-CoV-2 antiviral able to attenuate virus-induced inflammation
Source: Nat Commun. 2023 Jan 13;14:199. doi: 10.1038/s41467-023-35928-z (PMC9837764; doi:10.1038/s41467-023-35928-z)
Supplement: Supplementary file 3 — Reporting Summary [file 41467_2023_35928_MOESM3_ESM.pdf]

nature portfolio

Corresponding author(s): TMLS

Last updated by author(s): Jan 5, 2023

Reporting Summary

Nature Portfolio wishes to improve the reproducibility of the work that we publish. This form provides structure for consistency and transparency in reporting. For further information on Nature Portfolio policies, see our Editorial Policies and the Editorial Policy Checklist.

Statistics

For all statistical analyses, confirm that the following items are present in the figure legend, table legend, main text, or Methods section.

n/a	Confirmed	
☐	☒	The exact sample size (*n*) for each experimental group/condition, given as a discrete number and unit of measurement
☐	☒	A statement on whether measurements were taken from distinct samples or whether the same sample was measured repeatedly
☐	☒	The statistical test(s) used AND whether they are one- or two-sided<br>*Only common tests should be described solely by name; describe more complex techniques in the Methods section.*
☒	☐	A description of all covariates tested
☒	☐	A description of any assumptions or corrections, such as tests of normality and adjustment for multiple comparisons
☐	☒	A full description of the statistical parameters including central tendency (e.g. means) or other basic estimates (e.g. regression coefficient) AND variation (e.g. standard deviation) or associated estimates of uncertainty (e.g. confidence intervals)
☐	☒	For null hypothesis testing, the test statistic (e.g. *F*, *t*, *r*) with confidence intervals, effect sizes, degrees of freedom and *P* value noted<br>*Give P values as exact values whenever suitable.*
☒	☐	For Bayesian analysis, information on the choice of priors and Markov chain Monte Carlo settings
☒	☐	For hierarchical and complex designs, identification of the appropriate level for tests and full reporting of outcomes
☒	☐	Estimates of effect sizes (e.g. Cohen's *d*, Pearson's *r*), indicating how they were calculated

Our web collection on statistics for biologists contains articles on many of the points above.

Software and code

Policy information about availability of computer code

Data collection	Excel from Office 365 For windows 10
Data analysis	PrismGraphPad 9.0, Soft Max Pro 6.4 - Molecular Devices, Winnonlin Noncompartmental Analysis Program 8.2.0.4383 Core Version 11Oct2017, Pristima system (version 7.2; Xybion Medical System Co., USA), Mega 7.0 software, Spartan'18 software, PyMOL Delano Scientific LLC software (DeLano Scientific LLC; https://pymol.org/2/), on the usegalaxy.org site: FASTP v.0.20.1, BWA-MEM v. 0.7.17, SAMTools view v.1.13, iVar trim v.1.3.1, LoFreq v.2.1.5, iVar variants v.1.3.1, Pangolin v.3.1.17, Snpeff Version 5.1,. Mega 7.0 software, SinergyFinder2.0 (https://synergyfinder.fimm.fi/

For manuscripts utilizing custom algorithms or software that are central to the research but not yet described in published literature, software must be made available to editors and reviewers. We strongly encourage code deposition in a community repository (e.g. GitHub). See the Nature Portfolio guidelines for submitting code & software for further information.

Data

Policy information about availability of data

All manuscripts must include a data availability statement. This statement should provide the following information, where applicable:

- Accession codes, unique identifiers, or web links for publicly available datasets
- A description of any restrictions on data availability
- For clinical datasets or third party data, please ensure that the statement adheres to our policy

Replicates are presented in the graphics and source data files are available as online supporting materials. The source data file are provided supplementary files. The consensus sequencing data generated in this study have been deposited in the https://gisaid.org/ database under accession code: #EPI_ISL_1023783-EPI_ISL_1023845. The raw sequencing data on have been deposited in the bioproject PRJNA823058 (Severe acute respiratory syndrome coronavirus 2 (ID 823058) -

BioProject - NCBI (nih.gov)) and under SRA's (https://www.ncbi.nlm.nih.gov/sra) accession codes #SRR19181788-SRR19181796, SRR19181801, SRR191806-SRR19181809, SRR19181812 and SRR19181813.

Field-specific reporting

Please select the one below that is the best fit for your research. If you are not sure, read the appropriate sections before making your selection.

☒ Life sciences ☐ Behavioural & social sciences ☐ Ecological, evolutionary & environmental sciences

For a reference copy of the document with all sections, see nature.com/documents/nr-reporting-summary-flat.pdf

Life sciences study design

All studies must disclose on these points even when the disclosure is negative.

Sample size	Sample size was calculated to be at least the minimum necessary to perform statistical analysis.That is experiments were carried out at least three independent times, including a minimum of two technical replicates in each assay.
Data exclusions	Data exclusion was not applied
Replication	As experiments were carried out at least three independent times, the attempts to reproduce them were successful
Randomization	Animals were randomized using the random sequence generator available from http://www.random.org. Other experiments, on cell cullture-based and enzymatic experiments were not ramdomized
Blinding	The assays were performed blinded by one professional, codified and then read by another professional

Reporting for specific materials, systems and methods

We require information from authors about some types of materials, experimental systems and methods used in many studies. Here, indicate whether each material, system or method listed is relevant to your study. If you are not sure if a list item applies to your research, read the appropriate section before selecting a response.

Materials & experimental systems

n/a	Involved in the study	
☐	☒	Antibodies
☐	☒	Eukaryotic cell lines
☒	☐	Palaeontology and archaeology
☐	☒	Animals and other organisms
☐	☒	Human research participants
☒	☐	Clinical data
☒	☐	Dual use research of concern

Methods

n/a	Involved in the study	
☒	☐	ChIP-seq
☒	☐	Flow cytometry
☒	☐	MRI-based neuroimaging

Antibodies

Antibodies used	Anti-kinetin -Agrisera, AS09 444, 1/1000;<br>Anti-dsRNA - Jena Biosciences, RNT-SCI-10010200, 1/500;<br>Anti-human CD3 - BD Biosciences, 555342, 1/40;<br>Anti-human CD16-BD Biosciences, 561313, 1/20;<br>Rabbit IgG Isotype - BD Biosciences 550875, 1/25;<br>APC mouse IgG2a isotype - BD Biosciences, 550882, 1/40;<br>PE mouse IgG1 isotype - BD Biosciences, 555749, 1/20;<br>Peroxidase AffiniPure Goat Anti-Mouse IgG (H+L) - Jackson ImmunoResearch Inc, AB_10015289, 1:2000
Validation	Anti-kinetin – according to the manufacturer the Detection limit is 6 pg, 18 fmol, linear range of logit/log plot is 0,03-20 pmol, midrange (B/Bo =50 %) 120 pg, 340 fmol. As a control, experiments were performed in parallel with rabbit IgG isotype at the same dilution (mass/volume).<br>Anti-dsRNA and Peroxidase AffiniPure Goat Anti-Mouse IgG (H+L) were tested on mock and infected tissue to identify a dilution without unspecific staining in the uninfected control.<br>The other primary and secondary antibodies (Anti-human CD3, Anti-human CD16, APC mouse IgG2a isotype, PE mouse IgG1 isotype) were diluted based on products`s available datasheet information and in in-lab padronization before application.

Eukaryotic cell lines

Policy information about cell lines

Cell line source(s)	Calu-3, Huh-7 and VeroE6 cells. Calu-3 cell was donated by Farmanguinhos, cell culture platform RPT11M.: https://www.far.fiocruz.br/plataforma-de-avaliacao-de-atividade-antitumoral/. VeroE6 and Huh-7 cells were purchased from Rio de Janeiro's cell bank: https://bcrj.org.br/index.php. HEK293 cell line expressing recombinant human ERG potassium channel was purchased from BPS Bioscience (San Diego, CA, USA).
Authentication	We did not perform specific authentication on the cellular systems used
Mycoplasma contamination	Cells were periodically monitored as negative for mycoplasma using a PCR-based method
Commonly misidentified lines (See ICLAC register)	No Commonly misidentified lines were used according to the version 11, released 8 June 2021, on ICLAC website (https://iclac.org/databases/cross-contaminations/)

Animals and other organisms

Policy information about studies involving animals; ARRIVE guidelines recommended for reporting animal research

Laboratory animals	B6.Cg-Tg(K18-ACE2)2Prlmn/J - Purchased from https://www.jax.org/strain/034860 (Jackson Lab) and kept at Fiocruz (and used at 10-12 week-old). Golden hamsters were purchased at local pet shops and kept at Universidade Federal de Minas Gerais (and used at 6-8 week-old). CD-1 mice, Swiss mice and rats were also purchased from Charles River and mantained at Center of Innovation and Preclinical Studies (CIEnP). CD1 (6-8 week-old) and Swiss mice (5-10 week-old), and Sprague-Dawley rats (7-12 weeks-old), were used in the study. All experiments included Male and female animals. All animals were kept with 12h light/dark cycle with humidity of 50-58% and temperature of 29–30 °C.
Wild animals	No wild animals were used in the study
Field-collected samples	No field collected samples were used in the study.
Ethics oversight	Infection of K18-hACE2- mice were performed in the Animal Biosafety Level 3 (ABSL-3) multiuser facility, according to the animal welfare guidelines of the Ethics Committee of Animal Experimentation (CEUA-INCa, Licence 005/2021). Hamster infection was carried out in an ABSL-3 facility (ICB, UFMG), under Ethical approval by the Committee for Animal Experimentation of the UFMG (process no. 165/2021). Preclinical experiments in CD-1 mice, Swiss mice and Sprague-Dawley rats were performed in accordance to Ethics Committee approvals (CEUA # 210, 214, 215, 217, 241 and 306) of the Center of Innovation and Preclinical Studies (CIEnP).

Note that full information on the approval of the study protocol must also be provided in the manuscript.

Human research participants

Policy information about studies involving human research participants

Population characteristics	Healthy adult volunteers
Recruitment	To obtain human primary monocytes from peripheral blood mononuclear cells (PBMCs) we requested buffy coats from donors who decided to donate blood by spontaneous demand and signed the informed consent to the Hemotherapy Service of Hospital Clementino Fraga from the Federal University of Rio de Janeiro. All volunteers were de-identified.
Ethics oversight	The use of primary human cell was approved by Institutional Review Board from the Oswaldo Cruz Foundation under protocol 49971421.8.0000.5248 with signed informed consent in accordance with the Hemotherapy Service of Hospital Clementino Fraga from the Federal University of Rio de Janeiro. All volunteers were de-identified.

Note that full information on the approval of the study protocol must also be provided in the manuscript.
